# Mapping loci influencing blood pressure in the Framingham pedigrees using model-free LOD score analysis of a quantitative trait

**DOI:** 10.1186/1471-2156-4-S1-S74

**Published:** 2003-12-31

**Authors:** Jo Knight, Bernard V North, Pak C Sham, David Curtis

**Affiliations:** 1 Social, Genetic and Developmental Psychiatry Research Centre, Institute of Psychiatry, King's College, London, United Kingdom; 2 Joint Academic Department of Psychological Medicine, St. Bartholomew's and Royal London, School of Medicine and Dentistry, London, United Kingdom

## Abstract

This paper presents a method of performing model-free LOD-score based linkage analysis on quantitative traits. It is implemented in the QMFLINK program. The method is used to perform a genome screen on the Framingham Heart Study data. A number of markers that show some support for linkage in our study coincide substantially with those implicated in other linkage studies of hypertension.

Although the new method needs further testing on additional real and simulated data sets we can already say that it is straightforward to apply and may offer a useful complementary approach to previously available methods for the linkage analysis of quantitative traits.

## Background

Previously available methods of linkage analysis of quantitative traits applicable to complex pedigrees have consisted of variance components analysis [1-4], or regression analysis [5-7]. Although classical LOD score analysis of quantitative traits could be implemented using the original LINKAGE programs [8], doing so would have required full specification of the transmission model which would not generally be known. We have previously described a model-free LOD-score based method of linkage analysis applicable to dichotomous traits and implemented in the MFLINK program [9], and here we describe a development which allows its application to quantitative traits.

The Framingham data set includes genotypic data and phenotypic measures related to traits including blood pressure, characterized in a large set of complex pedigrees. Previously it has been investigated for linkage using the variance components method, producing a number of interesting results including a multipoint LOD score of 4.7 using a treatment-adjusted measure for blood pressure with markers on chromosome 17 [10]. Genome-wide linkage scans for blood pressure have also been carried out in a number of other data sets [11-16].

The present study describes the application of the new method of model-free linkage analysis of a quantitative trait to a measure of blood pressure obtained from the Framingham data set.

## Methods

The model-free method of linkage analysis that we present assumes that a susceptibility locus may have an effect on the trait in question such that a bi-allelic polymorphism exists with genotypes *AA*, *Aa*, and *aa*. The trait values for subjects having these genotypes have means M_AA_, M_Aa_, and M_aa_, with a constant variance of the measure around each genotype-specific mean V_AA _= V_Aa _= V_aa_. A model of heterogeneity is assumed, whereby the susceptibility locus exerts its effect in a proportion α of families while an unlinked locus exerts the same effect in the other families. For the purposes of analysis we assume equal frequencies for the two alleles and also constrain the genotype-specific means for the first two genotypes to be equal so that M_AA _= M_Aa_. This might be thought of as saying that the *A *allele exerts a dominant effect. The genotype *aa *with mean M_aa _is therefore less common, with frequency 0.25. Defining the overall population variance as V and the standard deviation as S = √V, then we set a variable T = (M_aa _- M_AA_)/S as a measure of the displacement between the genotype-specific means. There are limits for T at which the displacement between these means will be so large as to completely account for the overall population variance of the trait with zero variance around each mean (V_AA _= V_Aa _= V_aa _= 0), and between these limits it is straightforward to calculate a value for the variance around each genotype-specific mean (V_AA _= V_Aa _= V_aa_) that will produce the correct overall population variance, V. We allowed T to take a range of values ranging from -2 to 2. At the extreme values of M_aa _= M_AA _- 2S and M_aa _= M_AA _+ 2S the fraction of the overall variance explained by the difference in genotype-specific means is 75%, while this fraction decreases towards zero as the absolute value of S approaches zero. We set further parameters θ and α as follows. We write θ = t to imply the trait locus is at the test position, which may be on a multipoint map, and θ = 0.5 to indicate it is unlinked to any markers. We write α to be the proportion of families in which the trait locus exerts an effect, with the assumption that in proportion 1 - α of families an unlinked locus elsewhere exerts a similar effect. We then generate a range of transmission models by varying T from -2 through 0 to 2 and for each model we calculate three likelihoods which are functions of T, θ, and α:

L_UNLINKED _= L(θ = 0.5 or α = 0, T)

L_LINKED _= L(θ = t, α = 1, T)

L_HET _= L(θ = t, α, T) where L_HET _is maximized over α.

Three LOD scores are then defined as:

MLOD = log_10_(max [L_LINKED_/L_UNLINKED_]) - maximized over T

MALOD = log_10_(max [L_HET_/L_UNLINKED_]) - maximized over T and α

MFLOD = log_10_(max [L_LHET_]/max [L_UNLINKED_]) - numerator maximized over T and α, and denominator independently maximized over T.

These LOD scores are equivalent to those produced by the MFLINK program and the associated likelihood ratio statistics, calculated as 2ln(10)LOD (where ln(10) means the natural log of 10), are asymptotically distributed as chi-squared statistics as follows under the null hypothesis of the test locus exerting no effect on the quantitative trait (M_AA _= M_Aa _= M_aa _or α = 0). 2ln(10)MLOD is distributed as x ^2^_1_; 2ln(10)MALOD is distributed as a 50:50 mixture of x ^2^_2 _and x ^2^_0_; and 2ln(10)MFLOD as a 50:50 mixture of x ^2^_1 _and x ^2^_0_. The new method has been implemented in a computer program called QMFLINK.

The blood pressure trait was defined using a method similar to that used previously [10] except that it was not possible to correct for treatment effects because the relevant measures regarding response to treatment, which were derived from additional subjects not included in the sample, were not made available to us. Therefore, blood pressure measurements from subjects on treatment were dealt with in the same way as for those on no treatment. Data were extracted from the original cohort and the offspring cohort phenotype data sets. The mean systolic blood pressure was calculated over all measurements obtained at each visit attended by the subject for all subjects with at least 10 years between initial and final visit and who attended at least five visits in the original cohort and three visits in the offspring cohort. The mean body mass index (BMI) was also calculated, obtained from mean height and mean weight. For the two data sets and two genders, separate regression analyses were performed whereby the mean systolic blood pressure was regressed against BMI and age. The residuals were then standardized and used as a quantitative measure of systolic blood pressure corrected for BMI, age, gender, and cohort.

We carried out a genome scan using the corrected measure of systolic blood pressure by applying two-point model-free quantitative trait linkage analysis for each marker provided across the 22 chromosomes.

## Results

Table 1 displays the MLOD, MALOD, and MFLOD scores for all markers in which at least one of those values was statistically significant at ≤ 0.02 (as suggested by Rao et al. [17]). (All individual values that reached this level are in bold.) Positions in cM are those given by the Genetic Analysis Workshop 13 map files that are in turn derived from information on the Marshfield web site.

## Discussion

We compared our results with those from other linkage studies of hypertension [10-16]. Of particular interest were those published by Levy et al. [10] because they analyzed the same data set. All but one of the results supporting linkage to systolic blood pressure reported by this group were replicated in our own analysis. On chromosome 17 LOD scores of 3.8 and 3.1 at markers GATA25A04 and ATC6A06 reported by Levy et al. [10] correspond to the MLODs of 2.1 and 1.7, which have *p*-values of < 0.01 in our analysis. Furthermore, the marker GATA64A09 on chromosome 10, which has a LOD score of 2.0 in the study by Levy et al. [10] has an MLOD of 1.2 with a *p*-value of 0.02 in our analysis. The only result not replicated was their LOD of 2.0 on chromosome 5 at 59 cM, although we do have a marker with an MALOD of 1.4 (*p *= 0.02) at 20 cM.

A number of markers that we find to show some support for linkage seem to coincide with regions implicated in other linkage studies of hypertension. The distal region of chromosome 17 highlighted by our analysis has provided evidence of linkage in an affected sibling pair study by Julier et al. [18]. The positive results we detect with the more proximal markers at 24 cM and 49 cM on this chromosome could reflect the effect of the same gene as detected by Xu et al. [12] and Rutherford et al. [19], who both found evidence for linkage at 33 cM.

The MLOD of 1.3, MALOD of 1.4, and MFLOD of 0.9 (all *p *= 0.02) at 63 cM on chromosome 16 correspond with a region implicated to contain a low blood pressure QTL [12]. Finally, it is possible that the marker at 111 cM on chromosome 6 with an MLOD of 1.1 (*p *= 0.02) may reflect the effect of the same locus as was implicated in a study by Krushkal et al. [11], who found evidence for linkage significant at *p *= 0.0001 in the interval 141–153 cM.

We are not aware of any other published papers that report evidence of linkage to the specific regions on chromosomes 4, 5, and 8 where we have positive results. It is possible that the results from chromosome 5 and 8 as well as the result previously discussed result on chromosome 6 correspond to those from the MRC BRIGHT study [16] but at the time of writing only approximate locations for the putative QTLs highlighted by this study have been published.

## Conclusions

The new method of analysis has been applied to a large sample of complex pedigrees, some containing loops. The results overlap to some degree with those obtained using a variance-components method to analyze the same data set. However we could not obtain an identical phenotypic measure because we were unable to allow for treatment effects. Thus we do not know the extent to which differences in the methods of analysis and differences in quantitative measures account for differences between our results and those obtained in the previous analysis. It is of interest that our analysis also provides some evidence for linkage in areas that were not highlighted in the previous analysis, some at least of which seem to be supported by the results of other studies. Of course, we cannot be sure which results are true- or false-positives until the genetic basis of hypertension has been fully elucidated.

Analogously to the MFLINK program, the method can be described as "model-free" in that one does not need to specify in advance one particular inheritance model, but rather a range of models is considered. It is expected that if the true inheritance model is reasonably close to one of these then the method will be able to detect linkage when it is present, but if this were not the case then the method might lack sensitivity. Testing of MFLINK demonstrated that it had good ability to detect linkage under a variety of different conditions. The new method generates three different statistics, the MFLOD, MLOD, and MALOD. In general these will tend to be positively correlated but for a given marker one statistic may be more highly significant than the others. This could occur through chance or might happen when the test more accurately modelled the true biological situation. Having three partially independent statistics makes the overall interpretation of significance more problematic, but most users will probably wish to examine all three in order to avoid overlooking a potentially true positive result.

In order to determine the utility of the new method it will need to be thoroughly tested on a large number of real and simulated data sets. For now, we can say that the method is straightforward to apply and may offer a useful complementary approach to previously available methods for the linkage analysis of quantitative traits.

## Figures and Tables

**Table 1:** MLOD, MALOD, and MFLOD scores

**Chromosome**	**Locus Name**	**Position (cM)**	**MLOD^A^**	**MALOD^A^**	**MFLOD^A^**
4	GATA5B02	236	**2.0 (0.003)**	**2.2 (0.004)**	**1.3 (0.01)**
5	GATA3A04	20	0.7 (0.08)	**1.4 (0.02)**	0.7 (0.04)
6	GATA30A08	111	**1.1 (0.02)**	1.1 (0.04)	0.3 (NS)
8	GATA72C10	41	**1.6 (0.01)**	**1.6 (0.01)**	**0.9 (0.02)**
10	GATA64A09	137	**1.2 (0.02)**	1.2 (0.03)	0.5 (0.06)
16	GGAA3G05	64	**1.3 (0.02)**	**1.4 (0.02)**	**0.9 (0.02)**
17	GATA8C04	24	0.3 (NS)	**1.6 (0.01)**	0.2 (NS)
17	GATA185H04	49	**1.2 (0.02)**	1.2 (0.03)	0.0 (NS)
17	GATA25A04	67	**2.1 (0.002)**	**2.1 (0.004)**	0.7 (0.04)
17	ATC6A06	72	**1.7 (0.005)**	**1.7 (0.01)**	0.8 (0.03)

^A^Scores along with their associated *p*-values for all markers in which at least one of those values was statistically significant at ≤ 0.02.
